# Expanding potential targets of herbal chemicals by node2vec based on herb–drug interactions

**DOI:** 10.1186/s13020-023-00763-3

**Published:** 2023-06-01

**Authors:** Dai-yan Zhang, Wen-qing Cui, Ling Hou, Jing Yang, Li-yang Lyu, Ze-yu Wang, Ke-Gang Linghu, Wen-bin He, Hua Yu, Yuan-jia Hu

**Affiliations:** 1 State Key Laboratory of Quality Research in Chinese Medicine, Institute of Chinese Medical Sciences, University of Macau, 999078 Macao, China; 2 Shanxi Key Laboratory of Chinese Medicine Encephalopathy, Shanxi University of Chinese Medicine, Taiyuan, China; 3 DPM, Faculty of Health Sciences, University of Macau, Macao, China

**Keywords:** Node2vec, Traditional medicine, Herb–drug interaction, Link prediction, Cardiovascular disease

## Abstract

**Background:**

The identification of chemical–target interaction is key to pharmaceutical research and development, but the unclear materials basis and complex mechanisms of traditional medicine (TM) make it difficult, especially for low-content chemicals which are hard to test in experiments. In this research, we aim to apply the node2vec algorithm in the context of drug-herb interactions for expanding potential targets and taking advantage of molecular docking and experiments for verification.

**Methods:**

Regarding the widely reported risks between cardiovascular drugs and herbs, *Salvia miltiorrhiza* (Danshen, DS) and *Ligusticum chuanxiong* (Chuanxiong, CX), which are widely used in the treatment of cardiovascular disease (CVD), and approved drugs for CVD form the new dataset as an example. Three data groups DS-drug, CX-drug, and DS-CX-drug were applied to serve as the context of drug-herb interactions for link prediction. Three types of datasets were set under three groups, containing information from chemical-target connection (CTC), chemical-chemical connection (CCC) and protein–protein interaction (PPI) in increasing steps. Five algorithms, including node2vec, were applied as comparisons. Molecular docking and pharmacological experiments were used for verification.

**Results:**

Node2vec represented the best performance with average AUROC and AP values of 0.91 on the datasets “CTC, CCC, PPI”. Targets of 32 herbal chemicals were identified within 43 predicted edges of herbal chemicals and drug targets. Among them, 11 potential chemical-drug target interactions showed better binding affinity by molecular docking. Further pharmacological experiments indicated caffeic acid increased the thermal stability of the protein GGT1 and ligustilide and low-content chemical neocryptotanshinone induced mRNA change of FGF2 and MTNR1A, respectively.

**Conclusions:**

The analytical framework and methods established in the study provide an important reference for researchers in discovering herb–drug interactions, alerting clinical risks, and understanding complex mechanisms of TM.

**Supplementary Information:**

The online version contains supplementary material available at 10.1186/s13020-023-00763-3.

## Introduction

Due to changes in drug discovery patterns, classic reductionism was transformed into holism [1], and researchers turned their attention to the multi-effects of drugs, which called for exploring explicit targets of drugs to meet the requirements of complex analysis. The target identification of the drug is highly related to the associated therapies and side effects [2–4], which attracts a high level of attention. The traditional way to identify drug-target interactions is via biological experiments, which is relatively credible but time-consuming and costly [5]. With the development of computer science, some techniques were put forward to ease the experiment burden, such as molecular docking based on the three-dimensional structures of targets [6, 7], pharmacophore-based methods [6, 8], similarity searching [9], and machine learning [10, 11]. Under the guidance of theory, web applications and software were put forward to predict potential drug-target interactions, like Pharmmapper [12], the similarity ensemble approach [13], and TarFisdock [14]. In recent years, some new research applied artificial intelligence algorithms to explore potential targets, such as random forest, support vector machine [15], convolutional neural networks [16], and recurrent neural networks [17], which largely enriched the method of target prediction.

However, there is little research focusing on traditional medicine (TM). As reported, there are 75–80% of the world’s population [18] are users of TM, but its characteristic of “multi-compound, multi-target” makes it difficult in identifying biomolecules, especially the targets of TM chemicals, which further pose a great challenge on the effective usage and potential risk. For example, on the topic of the potential risk of drug-herb interactions [19, 20], widely used herbal medicines pose great risks to non-specialized practitioners, such as cardiovascular diseases (CVD) [21, 22]. At present, target identification of TM mainly relies on computer-based analysis tools developed from western drugs and experiments. Researchers in wet laboratories mainly focus on high-content chemicals and ignore other unmeasurable low-content ingredients, which may have cumulative effects. Therefore, TM needs specific new methods to expand potential targets of herbal chemicals without ignoring low-content chemicals.

In the research, a graph embedding algorithm called node2vec, inspired by Word2Vec [23], was proposed to explore the potential targets of TM. Node2vec can extract features from a graph and transform high-dimensional graph data into low-dimensional vector data [24, 25]. It has been applied in disease mechanism exploration [17] and drug-target interactions [26]. *Salvia miltiorrhiza* (Danshen, DS) and *Ligusticum chuanxiong* (Chuanxiong, CX) were chosen as examples in this study, which were widely used to treat conditions related to CVD [27, 28] by promoting blood circulation and removing blood stasis.

**Fig. 1:**
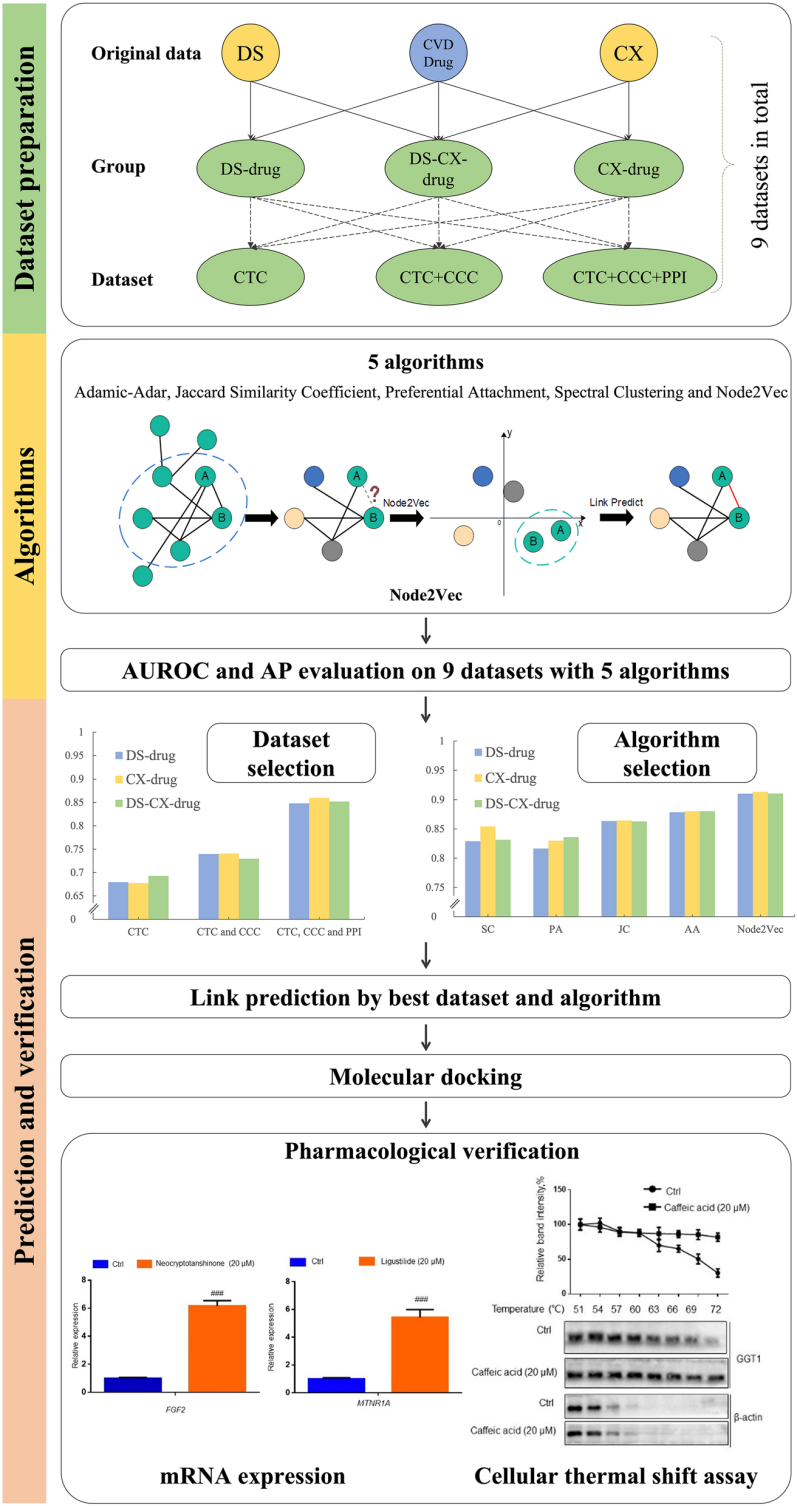
Research framework

Thus, this research aims to apply the node2vec algorithm in the context of drug–herb interactions for expanding potential targets of herbal chemicals by taking DS, CX and CVD-related approval drugs as examples and employing molecular docking and pharmacological experiments for verification. The research framework is clearly shown in Fig. 1. This study can provide an important reference for researchers to discover herb–drug interactions, alert clinical risks, and understand the complex mechanisms of TM.

## Methods and materials

### Data collection

#### Western drugs collection

To achieve reliable drug information related to CVD, we referred to the Preferred Reporting Items for Systematic Reviews and Meta-Analyses (PRISMA) [29]. The study retrieved drugs from the Drugbank database [30], and the Drugcentral database [31] served as a supplement. In detail, we filtered the database by ATC code with all subparts within the “C CARDIOVASCULAR SYSTEM” and two subparts, “B01 ANTITHROMBOTIC AGENTS” and “B02 ANTIHEMORRHAGICS,” within the “B BLOOD AND BLOOD FORMING ORGANS” to acquire CVD-related drugs. For these sampled drugs, we identified four aspects of drug information, including “Approval status,” “Known action,” “Organism,” and “CAS/SMILES,” to ensure the chosen drugs are still used in the market for humans with specific structures. One aspect of the drugs’ target information, “Uniprot ID of drug targets,” was used for target standardization.

#### Herbal chemicals collection

The chemical information of DS and CX was collected from the literature and three chemical databases: traditional Chinese Medicine System Pharmacology Database (TCMSP; http://tcmspw.com/tcmsp.php) [32]; Traditional Chinese Medicines Integrated Database (TCMID; http://www.megabionet.org/tcmid/) [33]; Shanghai Institute of Organic Chemistry of CAS Chemistry Database (http://www.organchem.csdb.cn) and The Encyclopedia of Traditional Chinese Medicine 2.0 (ETCM; http://www.tcmip.cn/ETCM2/front/#) [34, 35]. PubChem (http://pubchem.ncbi.nlm.nih.gov) [36] was used to standardize chemicals and supplement relevant chemical data, such as PubChem CID and SMILES information, and essential amino acids, monosaccharides, and disaccharides were excluded. To ensure the reliability of herbal chemical targets, we adopted “bioassay results” from PubChem, which showed detailed and credible activity information; only the results labeled as “active” in the activity column were chosen, and all targets were standardized by Uniprot (https://www.uniprot.org/) [37].

### Data processing and dataset preparation

#### Herb–drug interactions identification

Three kinds of interactions were involved, including the chemical-target connection (CTC), the similarity of chemicals (chemical–chemical connection, CCC), and the interaction of targets (protein–protein interactions, PPI). Firstly, the direct CTC was acquired during data collection after a strict screening process. Secondly, CCC was constructed by structural similarity analysis. The ChemmineR [38] toolkit running in R studio was used to perform a fingerprint-based chemical similarity search with a Tanimoto coefficient  ≥ 0.6 [39]. If the structural similarity of chemicals was 0.6 and above, two chemicals were connected. Thirdly, a PPI network was constructed to acquire PPI interactions by STRING [40], which can evaluate the tightness of proteins by providing a scoring system with a score range from 0 to 1. Only protein interaction scores of 0.9 and above were connected to ensure close target relationships.

#### Construction of groups, datasets and networks

The data on herbs and approved drugs formed different data groups. DS-drug and CX-drug groups were prepared directly. Meanwhile, because of their synergistic therapeutic effects clinically, DS and CX are regarded as the herbal pair to perform their function together. Therefore, the DS-CX-drug group was formed. These three groups were analyzed uniformly.

Among the three kinds of interactions, CTC is an indispensable part. Theoretically, structural similarity analysis and target interaction information will provide extra information and improve the accuracy of predictions, but comparisons are still needed. Therefore, due to the three types of interactions between chemicals and targets, three types of datasets were constructed to compare in every group. The first type of dataset only contained direct chemical and target interactions (CTC included). Considering the rule that structurally similar molecules have similar biological activities [41], information on chemical structure similarity was added for the second time (including CTC and CCC), and the third added correlations among proteins based on the second group for more information supplied (CTC, CCC, and PPI included). Finally, there are a total of nine datasets, three for each group.

### Link prediction

Identifying potential targets of TM complex systems is a key problem. Prof. Shao Li proposed the concept of network targets [42] to provide a theoretical basis for the solution of this problem. Li's team published successively for the mechanism of action of TCM prescriptions [43, 44], biomolecular markers of TCM evidence [45], etc. It is through networks that GE identifies potential targets in target prediction, and the concept of network targets provides theoretical support.

**Fig. 2:**
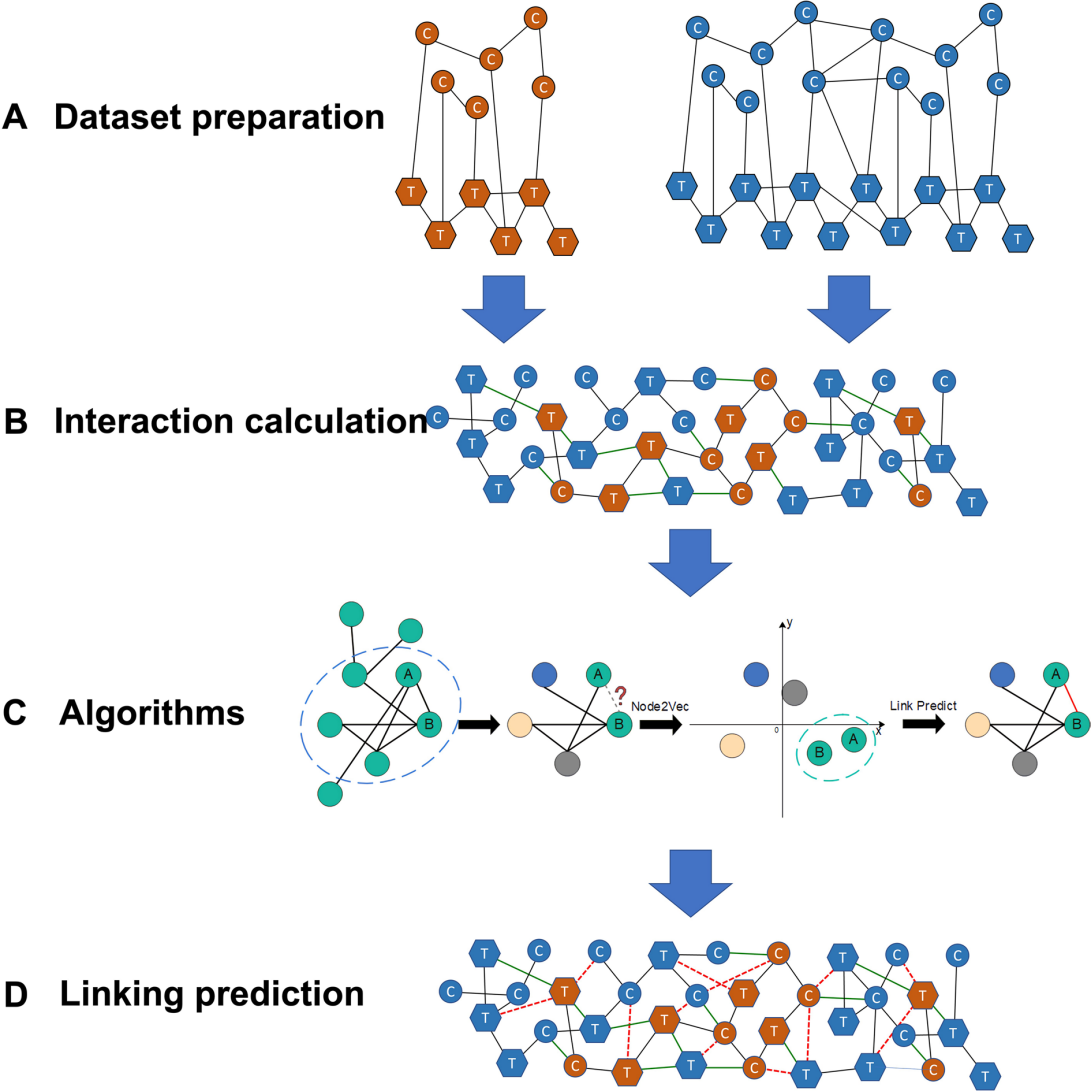
Diagram of methods of applying algorithms on link prediction. In processes **A**, **B** and **D**, chemicals of TM were colored in orange and approved drugs in blue. Circle nodes labeled with “C” meant chemicals and hexagons labeled with “T” meant targets. Black lines represented the connection between chemicals and targets from known knowledge. **A** meant the dataset from the curated database. In **B**, two individual networks were connected by integrating the links between chemicals and targets to form the CCT and PPI, which were labeled as green lines. **C** shown here mainly reflects the operating principle of node2vec. In **D**, new predicted interactions were labeled as red solid and dashed edges. In this research, we paid attention to the interaction between chemicals of TM and targets of approved drugs, which were labeled as red solid lines

In this study we transform the problem of target identification into link prediction, which is a method to predict the existence of a connection between two nodes. We selected one representation algorithm with node2vec of graph embedding (GE) and four traditional algorithms (Adamic-Adar, Jaccard similarity coefficient, preferential attachment, and spectral clustering) to evaluate how they perform on a chemical–target prediction task. We validated the results by checking the AP and the AUROC scores with tenfold cross validation. Each dataset is separated into a training set, a validation set, and a test set with a ratio of 6:3:1. A diagram of methods to explore potential targets were shown as Fig. 2 for better understanding. This diagram showed the dataset with CTC, CCC, PPI as an example to elucidate the process.

#### Algorithms

##### Node2vec

The node2vec algorithm, introduced by Aditya Grover and Jure Leskovec in 2016 [25], simply means transferring the data description of a node into a vector. Developed from DeepWalk [24], node2vec samples node information by random walk with bias. The basic idea of the algorithm is to form a low-dimensional vector space by extracting features from a graph by both a breadth-first search and a depth-first search. Node2vec applies two parameters to implement the strategy of random walk. Return Parameter ${\varvec{p}}$ controls the probability of the walk visiting a visited node, and a high value of ${\varvec{p}}$ tends to visit a node never before reached. In–Out Parameter ${\varvec{q}}$ controls the search visiting the base node inward or outward. After the data transformation step, a logistic regression algorithm is applied to the final classification task based on the vector-type data of a graph.

##### Adamic-Adar (AA)

The Adamic-Adar algorithm, a frequency weighted common neighbors algorithm, was introduced by Eytan Adar and Lada Adamic in 2003 [46]. The logarithmic function helps to create a weight to a shared neighbor between two nodes. This algorithm simply means that two nodes with more shared or common neighbors have more possibilities of linking.

It is defined as:

$${\varvec{AA}}_{{{\varvec{index}}\left( {{\varvec{A}},\user2{ B}} \right)}} = \user2{ }\mathop \sum \limits_{{{\varvec{Z}} \in \left( {{\varvec{N}}\left( {\varvec{A}} \right) \cap {\varvec{N}}\left( {\varvec{B}} \right)} \right)}} \frac{1}{{\log \left| {{\varvec{N}}\left( {\varvec{Z}} \right)} \right|}}$$

where ${\varvec{N}}\left({\varvec{x}}\right)$ is the set of neighbors connected to ${\varvec{x}}$.

##### Jaccard similarity coefficient (JS)

The Jaccard similarity coefficient algorithm was first introduced by Paul Jaccard and reformulated by Tanimoto TT [47]. This algorithm is commonly used to calculate the diversity or the similarity between two nodes.

The index is defined as:

$${\varvec{Jaccard}}_{{{\varvec{index}}\left( {{\varvec{A}},\user2{ B}} \right)}} = \user2{ }\frac{{\left| {{\varvec{N}}\left( {\varvec{A}} \right) \cap {\varvec{N}}\left( {\varvec{B}} \right)} \right|}}{{\left| {{\varvec{N}}\left( {\varvec{A}} \right) \cup {\varvec{N}}\left( {\varvec{B}} \right)} \right|}}$$

where ${\varvec{N}}\left({\varvec{x}}\right)$ is the set of neighbors connected to ${\varvec{x}}$.

##### Preferential attachment (PA)

The Preferential attachment algorithm was introduced in 1925 by Udny Yule and popularly applied in the Barabási–Albert model by Albert-László Barabási and Réka Albert. This algorithm considers that a node with more connected neighbors is more likely to have a new link.

It is defined as:

$${\varvec{PA}}_{{{\varvec{index}}\left( {{\varvec{A}},\user2{ B}} \right)}} = \user2{ }\left| {{\varvec{N}}\left( {\varvec{A}} \right)} \right| \times \left| {{\varvec{N}}\left( {\varvec{B}} \right)} \right|$$

where ${\varvec{N}}\left({\varvec{x}}\right)$ is the set of neighbors connected to ${\varvec{x}}$.

##### Spectral clustering (SC)

Spectral clustering, based on a normalized Laplacian matrix, belongs to the clustering algorithms family. It performs best when the original data is highly non-convex. Given an ${\varvec{n}}\times {\varvec{n}}$ adjacency matrix ${\varvec{A}}$ of the graph with ${\varvec{n}}$ nodes, a Laplacian matrix can be defined as:

$${\varvec{L}} = {\varvec{D}} - {\varvec{A}}$$

where ${\varvec{D}}$ is the ${\varvec{n}}\times {\varvec{n}}$ diagonal matrix of ${\varvec{A}}$.

After the data transformation step, Euclidean distance or k-nearest neighbors (KNN) algorithm will be applied on the Laplacian matrix with features from eigenvectors.

#### Evaluation

##### Average precision (AP) score

The AP score is one of a most popular and useful indicators on the prediction performance of a classification model. The score computes the Precision value ${\varvec{P}}$ while the Recall value ${\varvec{R}}$, a threshold for the metrics, increases from 0 to 1. The Precision value and the Recall value are defined as:

$${\varvec{Precision}} = \frac{{\user2{True \,Positive}}}{{\user2{True \,Positive} + \user2{False \,Positive}}}$$

$${\varvec{Recall}} = \user2{ }\frac{{\user2{True \,Positive}}}{{\user2{True\, Positive} + \user2{False \,Negative}}}$$

Once the Precision value and Recall value are calculated, the AP score can be computed by the equation given below:

$${\text{AP}} = \mathop \sum \limits_{{\varvec{n}}} \left( {{\varvec{R}}_{{\varvec{n}}} - {\varvec{R}}_{{{\varvec{n}} - 1}} } \right){\varvec{P}}_{{\varvec{n}}}$$

where ${{\varvec{R}}}_{{\varvec{n}}}$ and ${{\varvec{P}}}_{{\varvec{n}}}$ is the Recall value and the Precision value at the ***nth*** threshold.

##### Area under the receiver operating characteristic (AUROC) score

The AUROC score describes the expectation that a uniformly drawn random positive is ranked before a uniformly drawn random negative. It indicates precisely and comprehensively even if the dataset is imbalanced. The value varies from 0.5 to 1, as does the performance of the classification model from bad to good.

Before calculating the AUROC score, it is indispensable to draw a receiver operating characteristic (ROC) curve. A ROC curve consists of two parameters: true positive rate (TPR) and false positive rate (FPR). TPR is the same as the Recall value given above. FPR is defined as:

$${\varvec{FPR}} = \user2{ }\frac{{\user2{False \,Positive}}}{{\user2{False \,Positive} + \user2{True \,Negative}}}$$

The x-axis of a ROC curve is FPR, and the y-axis is TPR.

### Molecular docking

To verify the results of the GE link prediction, virtual molecular docking was used. The crystal structures of the targets were downloaded from the RCSB PDB (https://www.rcsb.org/) [48], and only X-ray structures with a resolution less than 3 Å were selected and saved as *pdb* format files. The ligand and receptor were split by Discovery Studio 4.5 [49]. Autodock Tools was used to prepare *pdbqt* format files. The gird boxes were adjusted to cover the entire pocket. After getting the related protein files, we searched the PubChem database for TM chemicals and Western drugs information, which were saved as *sdf* format and transformed into *pdbqt* format by OpenBabel to dock in the next step. Autodock Vina1.1.2 [50] was used to simulate the potential interactions among the selected chemicals and the targets.

### Experimental verification

Besides virtual molecular docking, cellular thermal shift assay (CETSA) and mRNA expression upon the treatment of predicated compounds were applied to verify predicted results.

#### Chemicals and reagents

Ginsenoside rb1, neocryptotanshinone, caffeic acid and ligustilide (the purities of all standards were higher than 98% by high-performance liquid chromatography analysis) were purchased from Chengdu Pufeide Biotech Co., Ltd. (Chengdu, China).

TRIzol™ Reagent, Fetal bovine serum (FBS), 0.25% Trypsin–EDTA (w/v), Dulbecco’s modified eagle's medium (DMEM), penicillin–streptomycin (10,000 U/mL, P/S), and phosphate-buffered saline (PBS) were purchased from Thermo Fisher Scientific (Waltham, MA, USA). Human MTNR1A polyclonal antibody and GGT1 polyclonal antibody were purchased from CLOUD-CLONE CORP. (CCC, USA). Anti-rabbit IgG, HRP-linked antibody was purchased from Cell Signaling Technology (Danvers, MA, USA). β-actin was purchased from COHESION BIOSCIENCES (SUZHOU, CHINA).

#### Cell culture

Human umbilical vein endothelial cells (HUVECs) were supplied by American Type Culture Collection (Manassas, Virginia, USA) and cultured in DMEM medium supplemented with 10% FBS and 1% P/S at 37 °C in an atmosphere of 95% humidity and 5% CO_2_. HUVECs were subjected to cell experiments when cultured to 90% confluence.

#### CETSA

The HUVECs cells were subcultured in a 100 mm cell culture dish and lysed with RIPA lysis buffer containing PMSF and protease inhibitor cocktail on ice for 10 min then centrifuged (12,000 × g, 10 min) at 4 ℃. Cell lysates were incubated with or without 20 μM compounds (Caffeic acid or Ligustilide) under shaking at 4 °C overnight. The protein concentration was adjusted to 2 μg/μL using RIPA lysis buffer. 40 μL cell lysates were transferred to new tubes and heated for 2.5 min for each tube at different temperatures (53–72 ℃) using a thermal mixer C (Eppendorf, USA). After centrifugation (12,000 × g for 10 min), 30 μL of the supernatants were incubated with 10 μL 5 × SDS-PAGE loading buffer at 95 ℃ for 10 min before western blotting assay.

### Quantitative real-time RT-PCR

Total RNA was extracted from HUVECs by TRIzol Reagent according to the manufacturer’s protocol. The content of total RNA was detected by the NanoVue spectrophotometer (Biochrom, United Kingdom). RNA was transcribed to cDNA using the PrimeScript™ RT Reagent Kit (TaKaRa Bio Inc., Kusatsu, Japan) by the manufacturer’s instruction. Real-time PCR was performed on a ViiA 7 Real-Time PCR System (Thermo Fisher Scientific, MA, USA). The primers were synthesized by IGE BIOTECHNOL OGY LTD (Guangzhou, China) and sequences were as follows: GGT1, forward TGACGTACCACCGCATCGTAGA and reverse CAGCGAAGAACTCGGAGGTCAT; MTNR1A, forward CTGGTCATCCTGTCGGTGTATC and reverse TCGACATCAGCACCAACGGGTA; β-actin, forward CACCATTGGCAATGAGCGGTTC and reverse AGGTCTTTGCGGATGTCCACGT.

The fold change of mRNA was determined relative to a blank control after normalizing to β-actin in each sample using the delta-delta Ct method.

## Results

### Data integration and screening

For western drugs, after screening the cardiovascular-related drugs by ATC code, we got 1203 drugs from two databases. Further screening processes were used to exclude data that did not meet the requirements, and 463 eligible drugs were acquired. We also excluded duplicate records, resulting in 378 drugs with different structures. Related targets of the filtered drugs were reserved.

782 herbal chemicals from three databases and literature without amino acids and simple saccharides were collected. After identifying their specific structures and related targets from the PubChem database, 117 were left. We excluded duplicate chemicals with the same structure and achieved 40 chemicals in DS and 38 chemicals in CX in the last step, which contained seven common chemicals.

**Fig. 3:**
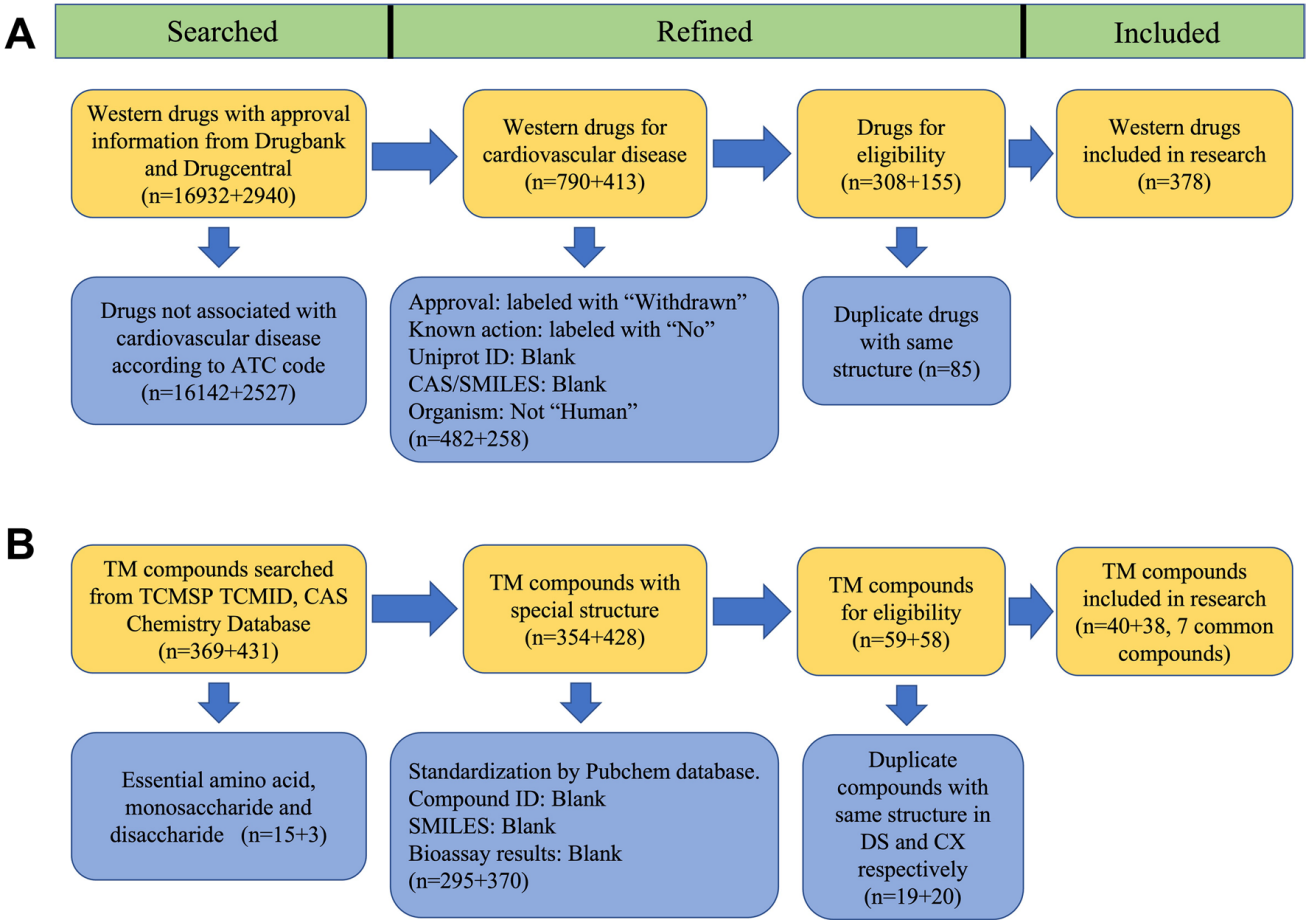
Flow diagram of filtered chemicals. The blue boxes showed the excluded chemicals with related reasons. **A** show the filtered Western drugs for CVD, and the two numbers in parentheses indicated the sources of Drugbank and Drugcentral, respectively. **B** showed the filtered herbal chemicals; the numbers in parentheses meant DS and CX, respectively

The filtering details of the drugs and herbal chemicals are shown in Fig. 3A and Fig. 3B.

### Group and dataset information

Based on herb–drug interactions, three herb–drug interaction groups were formed: DS-drug, CX-drug, and DS-CX-drug. In every group, three datasets were formed. As mentioned in the method section, to verify whether the extra structural similarity analysis and target interactions can help improve the accuracy of predictions, CTC, CTC & CCC, and CTC & CCC & PPI datasets were formed in each group. The statistical information was collected and integrated, as shown in Table 1.

**Table 1:** Statistical information of the three groups

Group	Node	Edge (dataset 1)	Edge (dataset 2)	Edge (dataset 3)
DS-drug	1497 (Chemical: 416 and target: 1081)	3429 (CTC: 3429)	4317 (CTC: 3429 and CCC: 888)	10562 (CTC: 3429 and CCC: 888 and PPI: 6245)
CX-drug	1435 (Chemical: 418 and target: 1017)	3311 (CTC: 3311)	4141 (CTC: 3311and CCC: 830)	9546 (CTC: 3311and CCC: 830 and PPI: 5405)
DS-CX-drug	1542 (Chemical: 449 and target: 1093)	3679 (CTC: 3679)	4619(CTC: 3679 and CCC: 940)	11001 (CTC: 3679 and CCC: 940 and PPI: 6382)

### Best dataset and algorithm selection

**Fig. 4:**
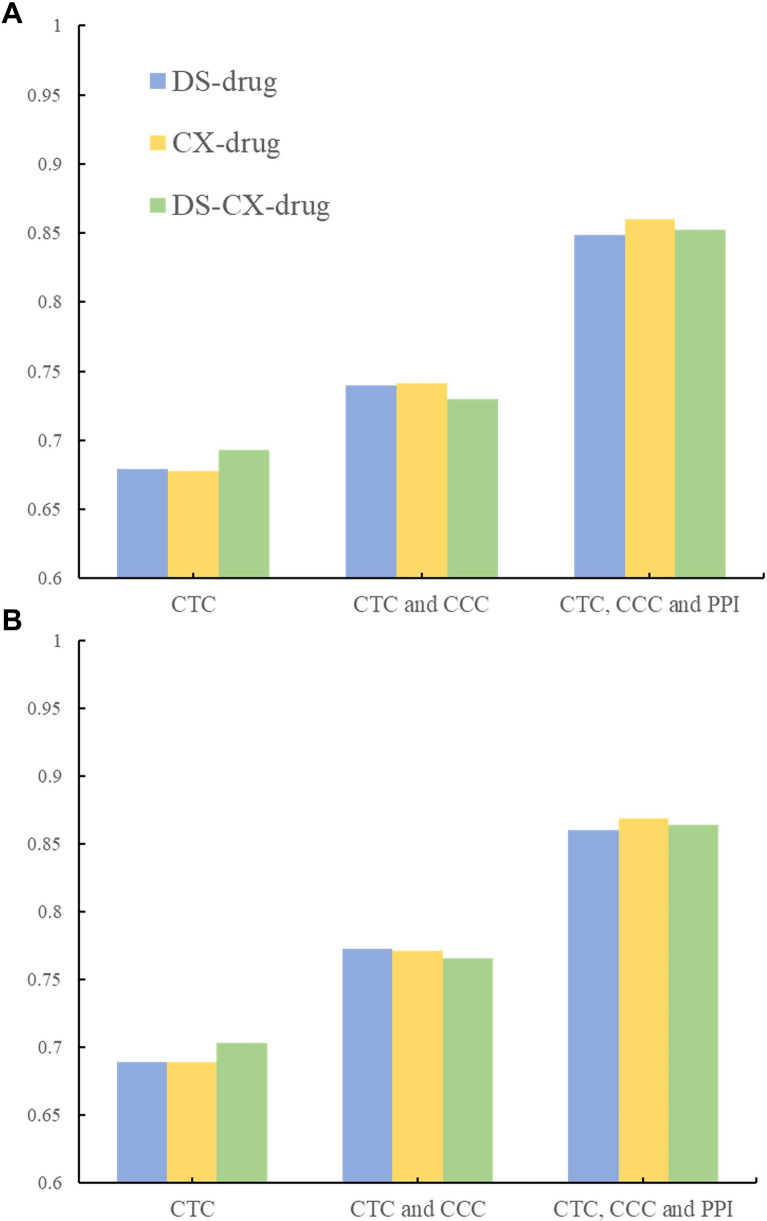
Average AUROC and AP scores of five algorithms in nine datasets. **A** and **B** showed AUROC and AP, respectively

We checked the AUROC and AP scores of nine datasets with five algorithms. To evaluate dataset performance and select the best one, the average AUROC and AP scores of five algorithms were calculated. As shown in Fig. 4, Compared to the other two types, the CTC & CCC & PPI datasets performed better in all three groups; that is, 0.86, 0.87, 0.86 with AUROC values, and 0.86, 0.85, 0.86 with AP values in DS-drug, CX-drug, and DS-CX-drug, respectively.

**Fig. 5:**
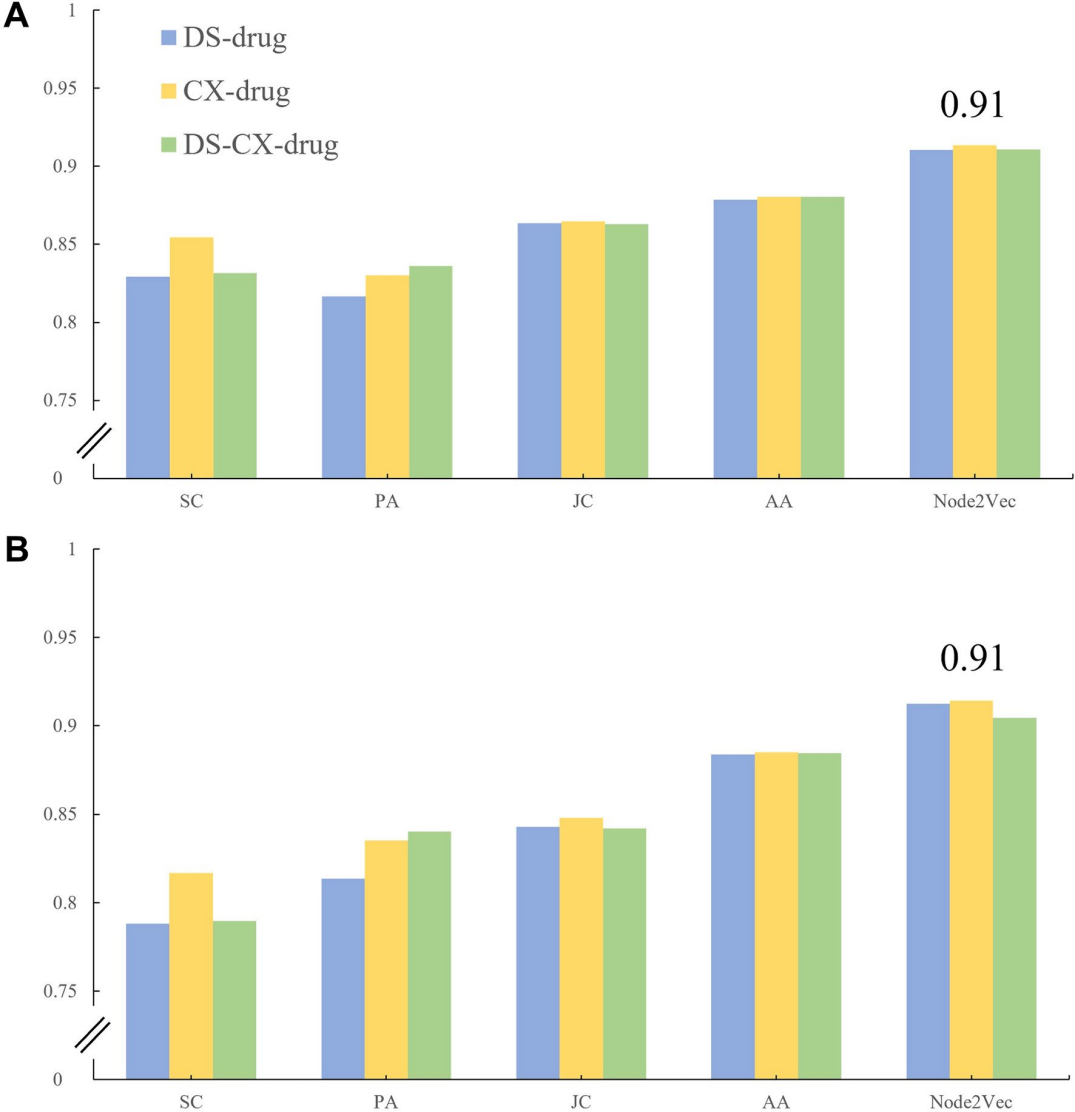
AUROC and AP of five algorithms in CTC & CCC & PPI datasets. **A** and **B** show AUROC and AP, respectively. Numbers of 0.91 show the average value of AUROC or AP

**Fig. 6:**
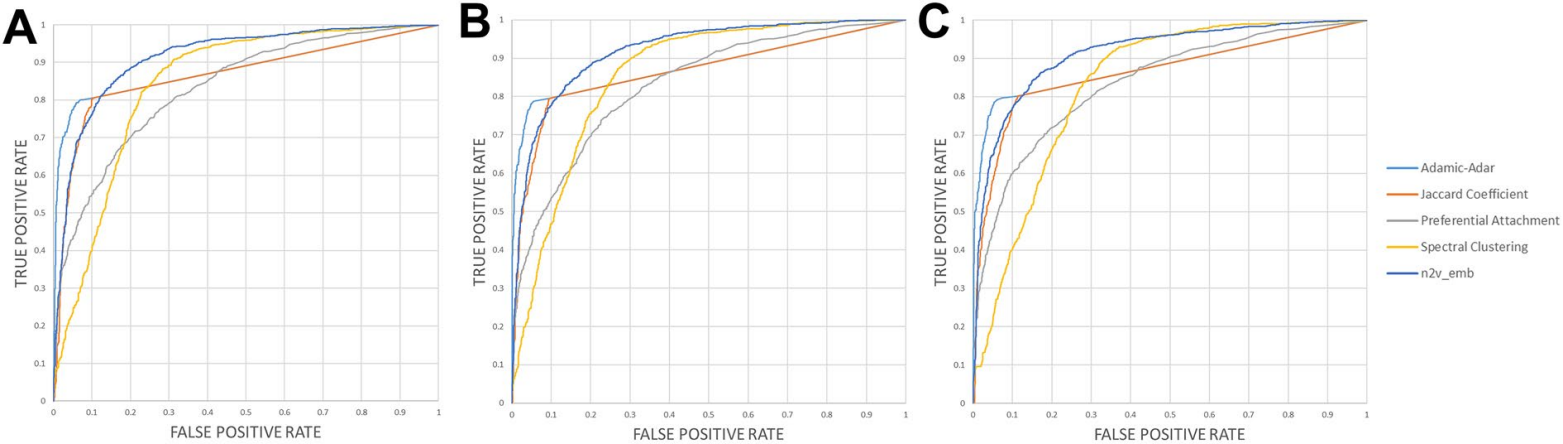
In three groups, the ROC curve of five algorithms on the CTC & CCC & PPI datasets. **A**, **B**, and **C** represent the groups of DS-drug, CX-drug and DS-CX-drug, respectively

On the other hand, node2vec and four other traditional algorithms were applied to compare and acquire link predictions of all datasets. We took a deeper look into the CTC & CCC & PPI dataset of each group and selected the best algorithm to further our research. As shown in Fig. 5, node2vec showed the best performance; that is, 0.91 with AUROC value, and 0.91, 0.91, 0.90 with AP values in the three groups, respectively. Furthermore, the ROC curve of the different algorithms intuitively on the CTC & CCC & PPI dataset in the three groups were shown in Fig. 6, which illustrated that the node2vec was better. For full data of AUROC and AP of all datasets, please check Additional file 1: Table S1 and Table S2. The datasets of CTC & CCC & PPI in three groups with average AUROC and AP values of 0.86 and the algorithm of node2vec with average AUROC and AP values of 0.91 were combined.

### Link prediction results

#### Expanded information

Based on the results of node2vec on the CTC & CCC & PPI datasets in the three groups, new predicted edges were counted and collected. Among predicted edges, we are just concerned about CTC. Furthermore, based on our research objectives, herbal chemical-drug target edges were the predicted information need to be counted. Besides, we were also concerned about how many herbal chemicals these predicted edges are associated with, which meant how many chemicals we provided new target information for. Hence, we counted the number of herbal chemical nodes. All information above were listed in Table 2. In DS-drug, CX-drug and DS-CX-drug groups, new edges of 445, 384, and 478 were respectively predicted. Compared to 236 herbal chemical-drug target edges of the whole CTC, targets of 32 herbal chemicals were identified within 43 predicted edges of herbal chemicals and drug targets. Detailed information of 43 predicted edges were listed in Additional file 1: Table S3

**Table 2:** Expansion information

Data group	Category	Original number	Expanded number 1
DS-drug	Total edges	10562	445 (4.21%)
	Herbal chemical-drug target edges	132	9 (6.82%)
	Total nodes	416	184 (44.23%)
	Herbal chemical nodes	40	20 (50.00%)
CX-drug	Total edges	9546	384 (4.02%)
	Herbal chemical-drug target edges	104	9 (8.65%)
	Total nodes	418	167 (39.95%)
	Herbal chemical nodes	38	11 (28.95%)
DS-CX-drug	Total edges	11001	478 (4.35%)
	Herbal chemical-drug target edges	236	25 (10.59%)
	Total nodes	449	142 (31.63%)
	Herbal chemical nodes	71	20 (28.17%)
All herbal chemical-drug target edges		236	43 (18.22%)
All chemical nodes		71	32 (45.07%)

1: The numbers in parentheses mean the expansion percentage in different categories

#### Further filtering of expanded information

The scope of data was further narrowed to select more appropriate data for verification. Firstly, we checked the indications of western drugs based on the collected targets. Although all drugs are related to CVDs by their ATC code, some drugs may contain multiple ATC codes whose main indication does not belong to CVDs. Taking Dexamethasone as an example, it has 16 ATC codes, including “C05 VASOPROTECTIVES,” but its main indication is for bacterial infections with inflammation in acute otitis media and acute otitis externa, which is not closely related to CVDs. Therefore, its target is not suitable as a potential target for CVD-related herbs. Secondly, we further excluded herbal chemicals without bioassay research or chemical profiling research from the TM published article to verify the importance of chemicals in TM. Thirdly, target structure information was checked using the PDB database, and SLC22A8 and POU2F2 were excluded due to an information shortage. Finally, 22 qualified CTC were chosen with 17 chemicals and 20 targets whose prediction values were 0.5 or above were filtered, including 12 CTC came from the DS-CX-drug group, 4 CTC from the DS-drug group, and 6 CTC from the CX-drug group. Low-content compounds were involved. In DS, the neocryptotanshinone, tanshindiol C of diterpene quinones, cyanidin of flavonoid metabolites and tigogenin of steroidal sapogenin were included. In CX, it includes three volatile oil components terpinolene, β-farnesene, and methyleugenol.

### Molecular docking

We searched the related X-ray structure of 22 targets. For ATP1B1, because it takes effect with ATP1A1, we docked chemicals with a complex of two proteins whose PDB ID is 3wgv. ATP1A2 and ATP1A1 are isoforms of the catalytic subunit, and they only exist in different organs. Due to ATP1A2 not having an adequate structure, we docked it by the structure of the ATP1A1-related protein complex with the same 3wgv structure. After that, 22 CTC including 20 targets and 17 chemicals were docked to show the binding affinity. Targets were also docked with their ligand and western drugs to compare them with the results of 22 CTC. As shown in Table 3, 11 CTC with better binding affinity than native ligands or drugs were listed. Complete docking information of all targets is presented in Additional file 1: Table S4

**Table 3:** The docking results of the herbal chemicals with investigated target proteins

Target	PDB entry	Chemicals	Chemical category	Binding Affinity (kcal/mol)	Group	Herb	Prediction value
ATP1A2	3wgv	Oligomycin A	Native ligand	− 9.9			
		Ouabain	Drug	− 7.3			
		Sitogluside	Herbal chemicals	− 7.3	DS-CX	DS	0.94
GGT1	6xpb	CU-6PMN	Native ligand	− 6.8			
		Aspirin	Drug	− 6			
		Caffeic acid	Herbal chemicals	− 6.2	CX	CX	0.93
CES2	1mx9	N-METHYLNALOXONIUM	Native ligand	− 8.5			
		Prasugrel	Drug	− 9.2			
		Neocryptotanshinone	Herbal chemicals	− 8.8	DS-CX	DS	0.93
CA1	1czm	3-Amabs	Native ligand	− 5.7			
		Chlorthalidone	Drug	− 9.6			
		3,4-Dihydroxybenzoic acid	Herbal chemicals	− 6	CX	CX	0.89
ATP1A2	3wgv	Oligomycin A	Native ligand	− 9.9			
		Ouabain	Drug	− 7.3			
		Ginsenoside rb1	Herbal chemicals	− 8.2	DS	DS	0.89
CSNK2A1	6yum	PQ8	Native ligand	− 9			
		Fostamatinib	Drug	− 9.3			
		Tanshindiol C	Herbal chemicals	− 9.7	DS	DS	0.82
MTNR1A	6me3	2-PHENYLMELATONIN	Native ligand	− 9.7			
		Dopamine	Drug	− 5.7			
		Ligustilide	Herbal chemicals	− 7.3	DS-CX	CX	0.81
FGF4	1ijt	Sulfate ion	Native ligand	− 2.6			
		Pentosan polysulfate	Drug	− 4.9			
		1-Pentadecanol	Herbal chemicals	− 3.2	DS-CX	CX	0.56
FGF2	5 × 1o	Inositol 1,4,5-Trisphosphate	Native ligand	− 5.9			
		Pentosan polysulfate	Drug	− 5.2			
		Neocryptotanshinone	Herbal chemicals	− 6.4	DS-CX	DS	0.51
SLC9A1	2ygg	Tris(Hydroxyethyl)aminomethane	Native ligand	− 3.2			
		Amiloride	Drug	− 4.3			
		Vitamin E	Herbal chemicals	− 4.5	DS-CX	DS	0.51
RPS6KA3	4nus	LJH685	Native ligand	− 10.1			
		Aspirin	Drug	− 5.9			
		Terpinolene	Herbal chemicals	− 6	CX	CX	0.50

### Experimental verification

#### CETSA indicated a direct interaction between GGT1 and caffeic acid

**Fig. 7:**
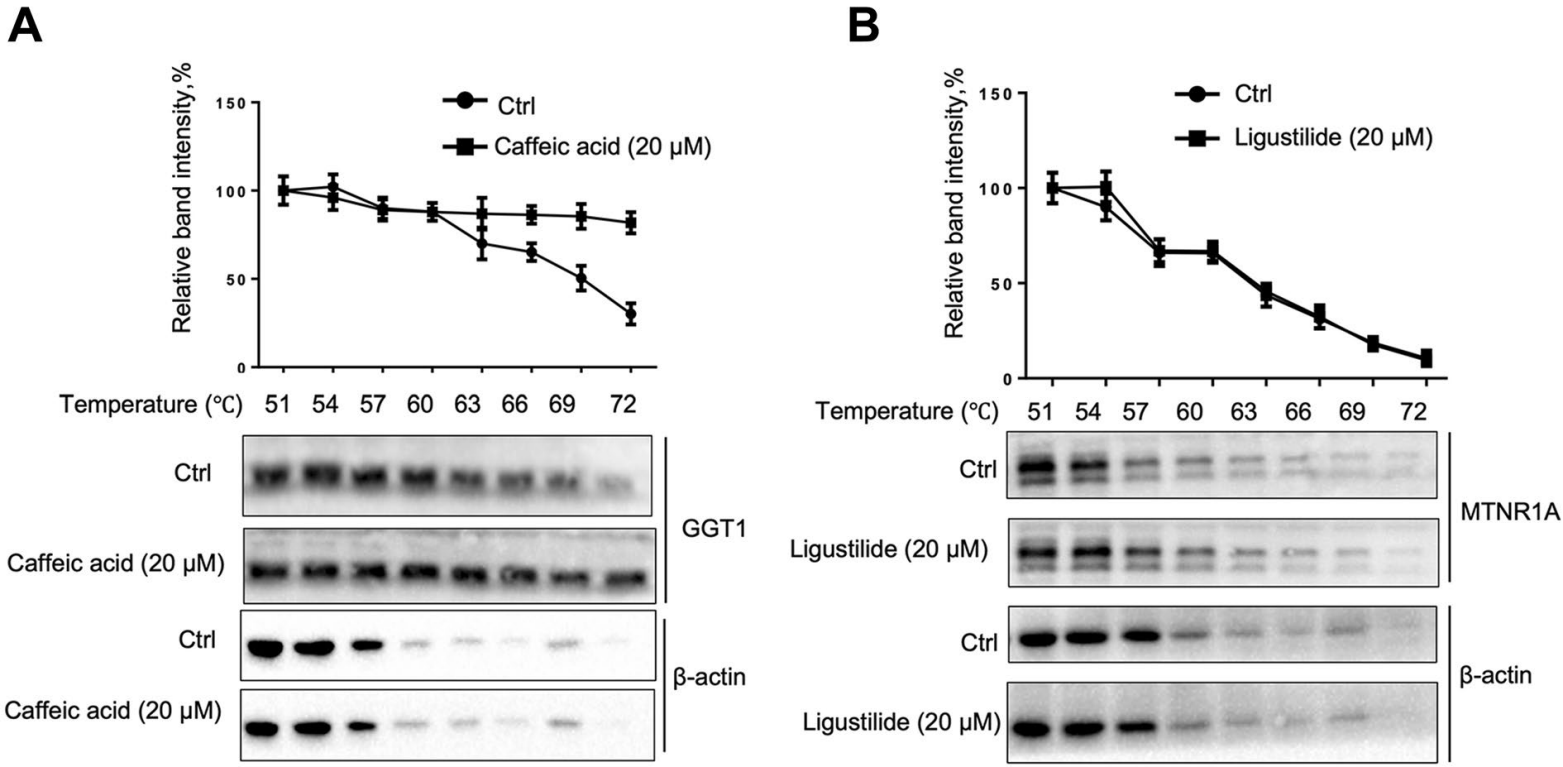
CETSA indicated caffeic acid increased the thermal stability of the GGT1 protein but ligustilide did not affect the thermal stability of the MTNR1A protein. CETSA was performed in HUVECs cell lysates after coincubation with 20 μM caffeic acid (**A**) or 20 μM ligustilide (**B**), then subjected to heating (51–72 ℃) before western blotting

CETSA is a valuable tool for the validation and optimization of drug target engagement [51]. CETSA showed that caffeic acid increased the thermal stability of the protein GGT1 (Fig. 7A), which indicated an interaction between caffeic acid and GGT1, suggesting GGT1 to be the direct target of caffeic acid. However, CETSA results showed that ligustilide did not interact with MTNR1A (Fig. 7B).

#### mRNA expression of GGT1, FGF2, CES2, MTNR1A, ATP1A2 upon the treatment of predicated compounds in the HUVECs

**Fig. 8:**
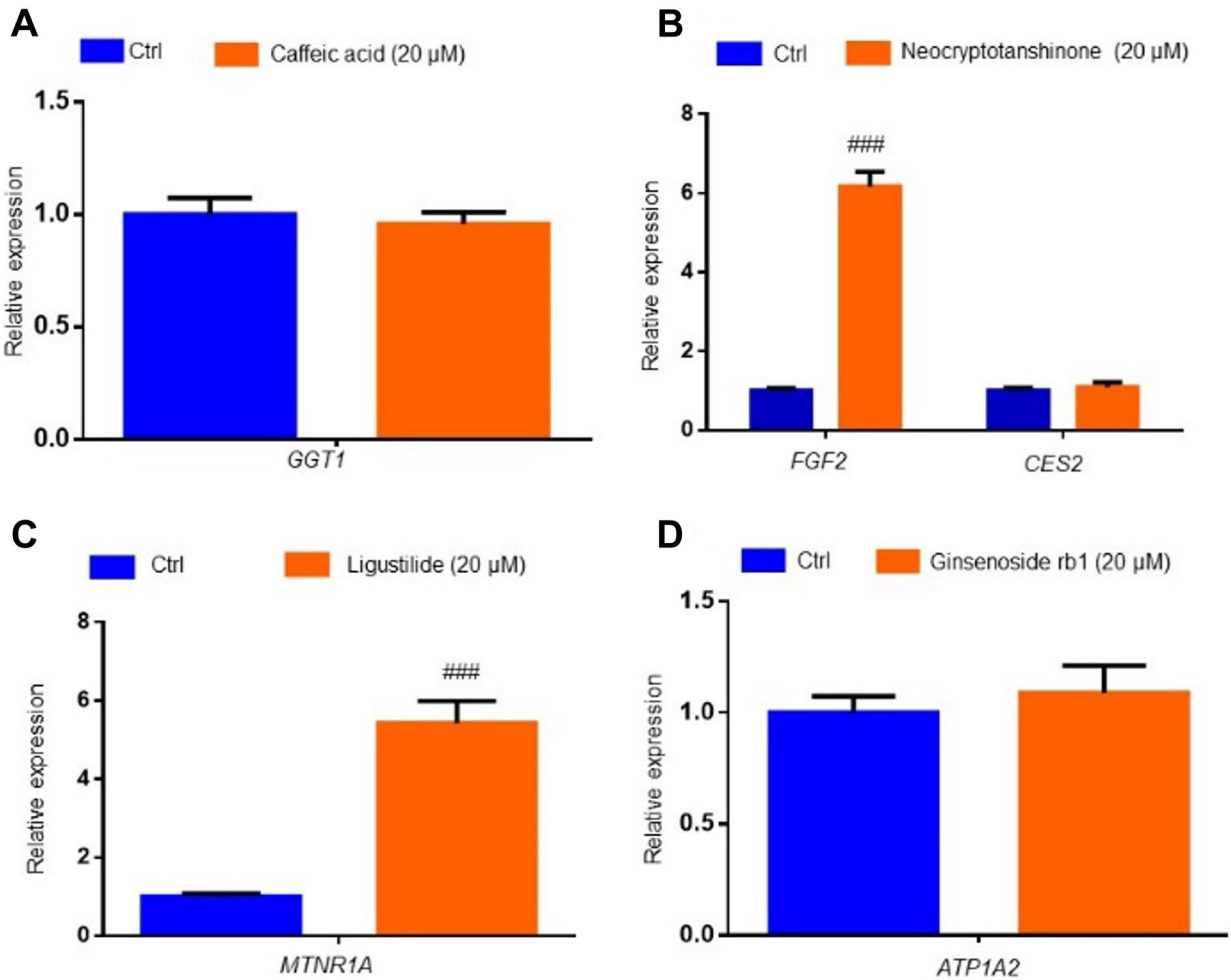
mRNA expression of GGT1, FGF2, CES2, MTNR1A, ATP1A2 upon the treatment of predicated compounds in the HUVECs. HUVECs were treated with 20 μM compounds of caffeic acid, neocryptotanshinone, ligustilide and ginsenoside rb1 for 6 h respectively, then subjected to a standard qPCR operation to determine the mRNA change of GGT1 (**A**), FGF2 (**B**), CES2 (**B**), MTNR1A (**C**), ATP1A2 (**D**) (n = 5). P^###^ < 0.001 vs the Ctrl group

Additionally, we detected the mRNA expression of GGT1, FGF2, CES2, MTNR1A, ATP1A2 upon 6 h treatment of corresponding compounds (caffeic acid, neocryptotanshinone, neocryptotanshinone, ligustilide, ginsenoside rb1) in the HUVECs. The experimental results showed that neocryptotanshinone (20 μM) and ligustilide (20 μM) induced the sixfold mRNA change of FGF2 (Fig. 8B) and 4.8-fold mRNA change of MTNR1A (Fig. 8C), respectively. The other three mRNA (GGT1, CES2, ATP1A2) did not change after the treatment of the corresponding compounds.

## Discussion

The exploration of drug-target interactions is always a hot and pivotal topic within modern drug development. Burdensome experiments cost money and time, but they do not produce the desired results [5]. Advances in computer technology are likely to compensate for this gap. Not only western drugs, but widely used and cheaper TM faces bigger questions because of its complex composition and mechanisms. Some artificial intelligence-based approaches have been used in the study of Chinese medicine, such as intelligent prescription recommendation systems [52]. However, few studies have been conducted to identify potential targets of natural compounds. In this research, a mature disease, CVD, was selected to conduct experiments on technology transfer exploration, which contains enough data on western drugs and curative TM. By integrating and screening data, multiple datasets and algorithms were set to choose the best combination. Finally, we expanded the dataset and acquired new potential targets for herbal chemicals.

From the beginning of the study design, we paid close attention to the reliability of the data. With a mature database, western drugs’ information is comprehensive with a high level of reliability. However, for the TM database, the situation is unsatisfactory. To pursue the reliability of TM data, a strict screening process results in a massive reduction in chemicals, which affects the subsequent analysis to some extent. Hence, the non-standardization and incomplete information in the TM database is a problem that requires urgent attention.

For the dataset scale, we chose approved CVD drug information as our background, which is not extensive but can be expanded in further research. On the one hand, other popular and widely used TM with good clinical efficacy can be merged with the drugs dataset to acquire predicted results, such as *Carthamus tinctorius* L [53]. and Angelica sinensis [54]. On the other hand, we can focus not only on CVD but also others. Otherwise, the type of data can be expanded from drugs to chemicals with better activity in vivo or in vitro.

We took CCC and PPI as supplements to CTC, which can be expanded. Some works showed interactions between biomolecules added by other information, such as the KGE model [55], which can integrate information on drug side effects, drug disease, protein disease, gene ontology annotation, and so on. Additionally, in the research on building credible negative samples to predict CTC [56], researchers still consider the protein data of sequence similarity and domain similarity. All of the above mentioned information can be transferred into the research of TM, but the data reliability of TM and its related information is still the key issue. One published work applied node2vec to TM [57], which combined different types of data. However, the research raises two questions. The first is that the herb target interactions in this article come from a TCM database that is not credible. The second is that consistency between the TCM indication and Western medicine theory was not clearly illustrated.

In algorithms, the performance of the model is highly correlated to the quality of the input dataset. A larger dataset carrying more information is more likely to contain the expected results. Managing a link prediction task is based on linked sample nodes and then masking those positive samples. In the case of an imbalanced dataset, however, there were a large number of unlinked nodes we could not take into consideration, which might be helpful to the model. Moreover, it could be possible to apply edge weight while processing embeddings in further work.

Back to the DS and CX in this research, these two herbs were chosen due to their wide research and application clinically and their huge sales in the TM market. For our research process and aims, two herbs have enough credible biological evidence to apply the new methods. Considering the common use of the two herbs, three groups were constructed to do the analysis, and the DS-CX-drug group contained full data of the other two groups. Therefore, the original predicted data from the DS-CX-drug group covered the other two. However, after the filtering process, the situation changed because different groups showed different prediction values about the same CTC, and only CTC with high values were left. One possible reason is the scale of the data. The scale of the DS-CX-drug is near twice the size of the other two, which causes prediction values to change. The other reason is the selection of filter values. The higher the tolerance, the higher the final similarity of DS-CX-drug and the other two. After link prediction, 17 compounds acquired new predicted potential targets, including 7 low-content chemicals. Molecular docking verified the interactions of herbal chemicals and drug targets, and half of them showed better binding affinity than native ligands or related drugs. In the experimental validation, caffeic acid increased the thermal stability of the protein GGT1, indicating direct interaction between caffeic acid and GGT1. GGT1 is a member of the Gamma-glutamyltransferase family. A large number of evidence suggests that elevated GGT activity is associated with an increased risk of CVD [58]. GGT was reported to be directly involved in atherosclerosis by promoting the atherosclerotic process, plaque instability and coronary ischemic events [59]. Ligustilide and low-content chemical neocryptotanshinone induced mRNA change of FGF2 and MTNR1A, respectively. FGF2 is associated with platelet and it can stimulate platelet-derived growth factors mRNA expression in a time-dependent and transient manner [60]. For MTNR1A, the receptor for melatonin, diabetes reduces its expression [61], and its ligand melatonin is associated with platelet activation and function [62]. This study combined virtual molecular docking, CETSA and mRNA expression means for partial validation, further and more robust in vivo and in vitro experimental studies are still to be developed.

The main role of this work is to identify potential targets of different components based on the multi-component systems of TM with available reliable data. The most relevant target is the exploration of the potential mechanism of the multicomponent system, which is the best application of the method for TM. The discovery of new potential targets has the possibility to unravel the mystery of the complex mechanisms that arise in some complex TM systems that produce good effects but are difficult to elucidate in some complex systems. In addition, the identification of activity-based quality control markers can also be applied to find potential targets for key compounds. In addition, the identification of activity-based quality control markers, formula composition, and herb–drug co-risk can be accomplished by identifying potential targets for key compounds through this method.

## Conclusions

After integrating and screening data, setting multiple datasets to acquire the best datasets and comparing algorithms, the CTC & CCC & PPI datasets with node2vec were selected for predictions. Based on the results, node2vec expands 43 edges of the herbal chemical-drug target based on the 236 original data. Compared to 71 herbal chemicals with different structures, it supplied new targets for 32 herbal chemicals, including low-content volatile oil or diterpenoids.

This study sufficiently expanded the potential target pool of herbal chemicals, including low-content chemicals that are hard to test by experimental approaches. This study employs a novel computational-based research framework that provides an important reference for researchers to understand herb–drug interactions, alarm potential clinical risks, and discover the complex mechanisms behind TM.

## Supplementary Information

**Additional file1: Table S1.** AUROC value of 5 algorithms applied on 9 datasets. **Table S2.** AP value of 5 algorithms applied on 9 datasets. **Table S3.** Predicted edges of herbal chemical-drug target. **Table S4.** Docking results.

## Data Availability

The datasets used and/or analysed during the current study are available from the corresponding author on reasonable request.

## Abbreviations

AA: Adamic-adar
AP: Average precision
AUROC: Area under the receiver operating characteristic
CCC: Chemical–chemical connection
CETSA: Cellular thermal shift assay
CTC: Chemical-target connection
CVD: Cardiovascular diseases
CX: Chuan xiong
DMEM: Dulbecco’s modified eagle's medium
DS: Dan shen
FBS: Fetal bovine serum
FPR: False positive rate
GE: Graph embedding
HUVECs: Human umbilical vein endothelial cells
JS: Jaccard similarity coefficient
PA: Preferential attachment
PBS: Phosphate-buffered saline
PPI: Protein–protein interactions
PRISMA: Preferred Reporting Items for Systematic Reviews
ROC: Receiver operating characteristic
SC: Spectral clustering
TM: Traditional medicine
TPR: True positive rate
