# Supplementary material for: Expanding potential targets of herbal chemicals by node2vec based on herb–drug interactions
Source: Chin Med. 2023 Jun 1;18:64. doi: 10.1186/s13020-023-00763-3 (PMC10233865; doi:10.1186/s13020-023-00763-3)
Supplement: Supplementary file 1 — Additional file1: Table S1. AUROC value of 5 algorithms applied on 9 datasets. Table S2. AP value of 5 algorithms applied on 9 datasets. Table S3. Predicted edges of herbal chemical-drug target. Table S4. Docking results. [file 13020_2023_763_MOESM1_ESM.docx]

**Supplementary tables**

Table S1. AUROC value of 5 algorithms applied on 9 datasets

| **Group** | **DS-drug** | | | **CX-drug** | | | **DS-CX-drug** | | |
| --- | --- | --- | --- | --- | --- | --- | --- | --- | --- |
| **Algorithms** | CTC | CTC, CCC | CTC, CCC, PPI | CTC | CTC, CCC | CTC, CCC, PPI | CTC | CTC, CCC | CTC, CCC, PPI |
| Adamic-Adar | 0.47 | 0.65 | 0.88 | 0.46 | 0.65 | 0.88 | 0.47 | 0.65 | 0.88 |
| Jaccard Coefficient | 0.47 | 0.64 | 0.86 | 0.46 | 0.63 | 0.86 | 0.47 | 0.64 | 0.86 |
| Preferential Attachment | 0.89 | 0.88 | 0.82 | 0.88 | 0.89 | 0.83 | 0.90 | 0.88 | 0.84 |
| Spectral Clustering | 0.76 | 0.77 | 0.83 | 0.77 | 0.80 | 0.85 | 0.79 | 0.78 | 0.83 |
| Node2Vec (Edge Embeddings) | 0.86 | 0.92 | 0.91 | 0.86 | 0.90 | 0.91 | 0.88 | 0.89 | 0.91 |
| **Average performance** | **0.69** | **0.77** | **0.86** | **0.69** | **0.77** | **0.87** | **0.70** | **0.77** | **0.86** |

Table S2. AP value of 5 algorithms applied on 9 datasets

| **Group** | **DS-drug** | | | **CX-drug** | | | | | **DS-CX-drug** | | |  |
| --- | --- | --- | --- | --- | --- | --- | --- | --- | --- | --- | --- | --- |
| **Algorithms** | CTC | CTC, CCC | CTC, CCC, PPI | | CTC | CTC, CCC | CTC, CCC, PPI | CTC | | CTC, CCC | CTC, CCC, PPI | |
| Adamic-Adar | 0.50 | 0.67 | 0.88 | | 0.50 | 0.67 | 0.88 | 0.50 | | 0.67 | 0.88 | |
| Jaccard Coefficient | 0.50 | 0.58 | 0.84 | | 0.50 | 0.56 | 0.85 | 0.50 | | 0.57 | 0.84 | |
| Preferential Attachment | 0.86 | 0.85 | 0.81 | | 0.87 | 0.86 | 0.84 | 0.89 | | 0.86 | 0.84 | |
| Spectral Clustering | 0.68 | 0.68 | 0.79 | | 0.67 | 0.72 | 0.82 | 0.71 | | 0.67 | 0.79 | |
| Node2Vec (Edge Embeddings) | 0.86 | 0.92 | 0.91 | | 0.85 | 0.90 | 0.91 | 0.87 | | 0.87 | 0.90 | |
| **Average performance** | **0.68** | **0.74** | **0.85** | | **0.68** | **0.74** | **0.86** | **0.69** | | **0.73** | **0.85** | |

Table S3. Predicted edges of herbal chemical-drug target

| **ID in dataset** | **Drug target** | **Predicted value** | **TM** | **Name** | **Group** |
| --- | --- | --- | --- | --- | --- |
| C015 | ESR2 | 0.954573 | CX | 1-Acetyl-2-phenylhydrazine | DS-CX |
| C071 | CES2 | 0.932791 | DS | 1-Hydroxy-2-[(2S)-1-hydroxypropan-2-yl]-8,8-dimethyl-6,7-dihydro-5H-phenanthrene-3,4-dione | DS-CX |
| C071 | FGF2 | 0.512451 | DS | 1-Hydroxy-2-[(2S)-1-hydroxypropan-2-yl]-8,8-dimethyl-6,7-dihydro-5H-phenanthrene-3,4-dione | DS-CX |
| C023 | ACHE | 0.93634462 | CX | 1-Pentadecanol | CX |
| C023 | FGF4 | 0.564913 | CX | 1-Pentadecanol | DS-CX |
| C023 | NR0B1 | 0.929076 | CX | 1-Pentadecanol | DS-CX |
| C010 | LEF1 | 0.854783 | CX | 2-Ethylphenol | DS-CX |
| C025 | TAOK1 | 0.511929 | CX | 2-Pentylfuran | DS-CX |
| C017 | TRPV3 | 0.9674303 | DS-CX | 3,4-Dihydroxybenzaldehyde | CX |
| C001 | CA1 | 0.89345833 | CX | 3,4-Dihydroxybenzoic acid | CX |
| C066 | SLC22A8 | 0.54502 | DS | beta-Caryophyllene oxide | DS-CX |
| C054 | GLUL | 0.50863743 | CX | beta-Farnesene | CX |
| C048 | GGT1 | 0.93457991 | CX | Caffeic acid | CX |
| C036 | PARP1 | 0.78834827 | DS | Cryptotanshinone | DS |
| C034 | PDE4B | 0.543216 | DS | Cyanidin | DS-CX |
| C044 | SLC12A1 | 0.61130703 | CX | Ferulic acid | CX |
| C012 | HDAC8 | 0.602179 | CX | Furfural | DS-CX |
| C067 | ATP1A2 | 0.88670764 | DS | Ginsenoside rb1 | DS |
| C067 | ATP1B1 | 0.993371 | DS | Ginsenoside rb1 | DS-CX |
| C049 | RXRA | 0.957055 | DS | Isoferulic acid | DS-CX |
| C029 | SRD5A2 | 0.67389809 | DS | Isoimperatorin | DS |
| C061 | MME | 0.585547 | CX | Ligustilide | DS-CX |
| C061 | MTNR1A | 0.814885 | CX | Ligustilide | DS-CX |
| C070 | POU2F2 | 0.53773731 | DS | Methyl tanshinonate | DS |
| C011 | MDH2 | 0.677913 | CX | Methyleugenol | DS-CX |
| C059 | CHRM5 | 0.61610662 | DS | Oleyl alcohol | DS |
| C021 | IDE | 0.845555 | DS | Physcion | DS-CX |
| C007 | IL5 | 0.56495172 | DS | Salsalate | DS |
| C064 | ATP1A2 | 0.944102 | DS | Sitogluside | DS-CX |
| C008 | TTK | 0.55345753 | DS-CX | Stearic acid | DS |
| C033 | CSNK2A1 | 0.81818293 | DS | Tanshindiol C | DS |
| C033 | HDAC8 | 0.871818 | DS | Tanshindiol C | DS-CX |
| C022 | RPS6KA3 | 0.50171901 | CX | Terpinolene | CX |
| C031 | ATP1B1 | 0.71045817 | DS | Tigogenin | DS |
| C014 | DRD1 | 0.7557824 | CX | Undecanoic acid | CX |
| C014 | NR3C1 | 0.66502273 | CX | Undecanoic acid | CX |
| C014 | SLC27A2 | 0.638596 | CX | Undecanoic acid | DS-CX |
| C014 | SLC2A5 | 0.610588 | CX | Undecanoic acid | DS-CX |
| C016 | AR | 0.740133 | DS | Veratraldehyde | DS-CX |
| C016 | CYP2C18 | 0.782496 | DS | Veratraldehyde | DS-CX |
| C016 | MDH2 | 0.903922 | DS | Veratraldehyde | DS-CX |
| C024 | ORM2 | 0.640584 | DS | Vitamin E | DS-CX |
| C024 | SLC9A1 | 0.508962 | DS | Vitamin E | DS-CX |

Table S4. Docking results

| **Target** | **PDB entry** | **Chemicals** | **Category** | **Binding Energy** | **Dataset** | **TCM** | **Prediction value** |
| --- | --- | --- | --- | --- | --- | --- | --- |
| ATP1B1 | 3wgv | Oligomycin A | Native ligand | -9.9 | - | - | - |
|  |  | Digoxin | Drug | -11 | - | - | - |
|  |  | Ginsenoside rb1 | TM compounds | -8.2 | DS-CX | DS | 0.99 |
| RXRA | 6jno | CU-6PMN | Native ligand | -10.6 | - | - | - |
|  |  | Bezafibrate | Drug | -8.2 | - | - | - |
|  |  | Isoferulic acid | TM compounds | -6.6 | DS-CX | DS | 0.96 |
| ATP1A2 | 3wgv | Oligomycin A | Native ligand | -9.9 | - | - | - |
|  |  | Ouabain | Drug | -7.3 | - | - | - |
|  |  | Sitogluside | TM compounds | -7.3 | DS-CX | DS | 0.94 |
| ACHE | 6wv1 | HI-6 | Native ligand | -8.3 | - | - | - |
|  |  | Ephedrine | Drug | -6.3 | - | - | - |
|  |  | 1-Pentadecanol | TM compounds | -6 | CX | CX | 0.94 |
| GGT1 | 6xpb | CU-6PMN | Native ligand | -6.8 | - | - | - |
|  |  | Aspirin | Drug | -6 | - | - | - |
|  |  | Caffeic acid | TM compounds | -6.2 | CX | CX | 0.93 |
| CES2 | 1mx9 | N-METHYLNALOXONIUM | Native ligand | -8.5 | - | - | - |
|  |  | Prasugrel | Drug | -9.2 | - | - | - |
|  |  | Neocryptotanshinone | TM compounds | -8.8 | DS-CX | DS | 0.93 |
| CA1 | 1czm | 3-Amabs | Native ligand | -5.7 | - | - | - |
|  |  | Chlorthalidone | Drug | -9.6 | - | - | - |
|  |  | 3,4-Dihydroxybenzoic acid | TM compounds | -6 | CX | CX | 0.89 |
| ATP1A2 | 3wgv | Oligomycin A | Native ligand | -9.9 | - | - | - |
|  |  | Ouabain | Drug | -7.3 | - | - | - |
|  |  | Ginsenoside rb1 | TM compounds | -8.2 | DS | DS | 0.89 |
| CSNK2A1 | 6yum | PQ8 | Native ligand | -9 | - | - | - |
|  |  | Fostamatinib | Drug | -9.3 | - | - | - |
|  |  | Tanshindiol C | TM compounds | -9.7 | DS | DS | 0.82 |
| MTNR1A | 6me3 | 2-PHENYLMELATONIN | Native ligand | -9.7 | - | - | - |
|  |  | Dopamine | Drug | -5.7 | - | - | - |
|  |  | Ligustilide | TM compounds | -7.3 | DS-CX | CX | 0.81 |
| ATP1B1 | 3wgv | Oligomycin A | Native ligand | -9.9 | - | - | - |
|  |  | Digoxin | Drug | -11 | - | - | - |
|  |  | Tigogenin | TM compounds | -8.1 | DS | DS | 0.71 |
| MDH2 | 4wlu | Nicotinamide_dinucleotide | Native ligand | -10.2 | - | - | - |
|  |  | Xanthinol | Drug | -6.2 | - | - | - |
|  |  | Methyleugenol | TM compounds | -5.4 | DS-CX | CX | 0.68 |
| ORM2 | 3apw | Disopyramide | Native ligand | -8.7 | - | - | - |
|  |  | Disopyramide | Drug | -8.7 | - | - | - |
|  |  | Vitamin E | TM compounds | -8.5 | DS-CX | DS | 0.64 |
| SLC12A1 | 6nph | SCHEMBL1649042 | Native ligand | -7.1 | - | - | - |
|  |  | Torasemide | Drug | -8.1 | - | - | - |
|  |  | Ferulic acid | TM compounds | -6.1 | CX | CX | 0.61 |
| MME | 2yb9 | Heteroarylalanine 5-Phenyl Oxazole | Native ligand | -9.8 | - | - | - |
|  |  | Sacubitril | Drug | -8.9 | - | - | - |
|  |  | Ligustilide | TM compounds | -6.2 | DS-CX | CX | 0.59 |
| FGF4 | 1ijt | SULFATE ION | Native ligand | -2.6 | - | - | - |
|  |  | Pentosan polysulfate | Drug | -4.9 | - | - | - |
|  |  | 1-Pentadecanol | TM compounds | -3.2 | DS-CX | CX | 0.56 |
| TTK | 7chm | Compound CID: 155907519 | Native ligand | -10.6 | - | - | - |
|  |  | Fostamatinib | Drug | -6 | - | - | - |
|  |  | Stearic acid | TM compounds | -5.8 | DS | DS-CX | 0.55 |
| PDE4B | 1xm4 | Piclamilast | Native ligand | -9.5 | - | - | - |
|  |  | Iloprost | Drug | -8.7 | - | - | - |
|  |  | Cyanidin | TM compounds | -8.5 | DS-CX | DS | 0.54 |
| FGF2 | 5x1o | Inositol 1,4,5-Trisphosphate | Native ligand | -5.9 | - | - | - |
|  |  | Pentosan polysulfate | Drug | -5.2 | - | - | - |
|  |  | Neocryptotanshinone | TM compounds | -6.4 | DS-CX | DS | 0.51 |
| SLC9A1 | 2ygg | TRIS(HYDROXYETHYL)AMINOMETHANE | Native ligand | -3.2 | - | - | - |
|  |  | Amiloride | Drug | -4.3 | - | - | - |
|  |  | Vitamin E | TM compounds | -4.5 | DS-CX | DS | 0.51 |
| GLUL | 2ojw | ADENOSINE-5'-DIPHOSPHATE | Native ligand | -13 | - | - | - |
|  |  | Diazoxide | Drug | -6.8 | - | - | - |
|  |  | beta-Farnesene | TM compounds | -6.2 | CX | CX | 0.51 |
| RPS6KA3 | 4nus | LJH685 | Native ligand | -10.1 | - | - | - |
|  |  | Aspirin | Drug | -5.9 | - | - | - |
|  |  | Terpinolene | TM compounds | -6 | CX | CX | 0.50 |
